# Characteristics of brain topological networks in posterior cortical atrophy: a hybrid FDG PET/MRI study

**DOI:** 10.1002/alz.090272

**Published:** 2025-01-09

**Authors:** Liyong Wu, Min Chu, Binbin Nie, Li Liu

**Affiliations:** ^1^ Xuanwu Hospital, Capital Medical University, Beijing China; ^2^ Beijing Engineering Research Center of Radiographic Techniques and Equipment, Institute of High Energy Physics, Chinese Academy of Sciences, Beijing China

## Abstract

**Background:**

Graph theory is an advanced method for analyzing the balance of brain networks. However, the changes in white matter (WM) and metabolic networks and their correlation with clinical features in patients with posterior cortical atrophy (PCA) require further investigation. This study aims to clarify the structural, metabolic, WM, and metabolic topological network in PCA, and explore their correlation with clinical features.

**Method:**

Thirty‐four PCA patients and 34 healthy controls (HC) participated in the study and underwent hybrid FDG‐PET/MRI scans and neuropsychological evaluations. Voxel‐based morphometric analysis and Tract‐Based Spatial Statistics was used to assess gray matter volume and white matter integrity and standard uptake value ratio (SUVR) was calculated to evaluate the gray matter metabolism. Then, the topological network connectivity of 90 brain regions was computed using graph theory analysis.

**Result:**

PCA patients exhibited gray matter atrophy and metabolic decline in the posterior regions (including the occipital, temporal, and parietal lobes), while white matter damage was more widespread, affecting the parietal, temporal and frontal to occipital tract. In both WM and metabolic topological network analyses, PCA patients displayed significant damage to global and local topological network properties. Particularly in local properties, including nodal centrality, degree centrality, nodal clustering coefficient, nodal efficiency, and nodal shortest path, the changes in the metabolic network were more significant than those in the white matter network. Not only were the posterior brain regions affected, but the frontal node was significantly impacted. PCA patients' WM and metabolic topological networks exhibited different centrality distribution patterns. Several network metrics were correlated with the clinical severity of PCA and the degree of visual impairment, notably the close association between the occipito‐frontal network and visual functional impairment.

**Conclusion:**

The study revealed complex alterations in the topological brain networks in PCA patients, not only confined to the posterior brain but also involving the frontal nodes. In addition, the brain topological network characteristics can serve as important markers for assessing the severity of PCA, with potential value for earlier diagnosis and therapeutic intervention.

